# Case Report: VEXAS Syndrome: From Mild Symptoms to Life-Threatening Macrophage Activation Syndrome

**DOI:** 10.3389/fimmu.2021.678927

**Published:** 2021-04-23

**Authors:** Frederik Staels, Albrecht Betrains, F. J. Sherida H. Woei-A-Jin, Nancy Boeckx, Marielle Beckers, An Bervoets, Mathijs Willemsen, Barbara Neerinckx, Stephanie Humblet-Baron, Daniel Engelbert Blockmans, Steven Vanderschueren, Rik Schrijvers

**Affiliations:** ^1^Laboratory of Adaptive Immunology, Immunology and Transplantation, Department of Microbiology, KU Leuven, Leuven, Belgium; ^2^Allergy and Clinical Immunology Research Group, Immunology and Transplantation, Department of Microbiology, KU Leuven, Leuven, Belgium; ^3^Department of General Internal Medicine, University Hospitals Leuven, Leuven, Belgium; ^4^Laboratory of Clinical Infectious and Inflammatory Disease, Immunology and Transplantation, Department of Microbiology, KU Leuven, Leuven, Belgium; ^5^Department of General Medical Oncology, Leuven Cancer Institute, University Hospitals Leuven, Leuven, Belgium; ^6^Laboratory of Experimental Oncology, Department of Oncology, KU Leuven, Leuven, Belgium; ^7^Clinical Department of Laboratory Medicine, University Hospitals Leuven, Leuven, Belgium; ^8^Laboratory of Experimental Hematology, Department of Oncology, KU Leuven, Leuven, Belgium; ^9^Department of Hematology, University Hospitals Leuven, Leuven, Belgium; ^10^Department of Dermatology, University Hospitals Leuven, Leuven, Belgium; ^11^Department of Dermatology, University Hospitals Antwerpen, Edegem, Belgium; ^12^VIB-KU Leuven Center for Brain and Disease Research, KU Leuven, Leuven, Belgium; ^13^Department of Rheumatology, University Hospitals Leuven, Leuven, Belgium; ^14^Skeletal Biology and Engineering Research Center, Department of Development and Regeneration, KU Leuven, Leuven, Belgium

**Keywords:** primary immunodeficiencies, autoinflammation, treatment reactions, myelodysplasia, ubiquitination

## Abstract

Recently, a novel disorder coined VEXAS (vacuoles, E1 enzyme, X-linked, autoinflammatory, somatic) syndrome was identified in patients with adult-onset inflammatory syndromes, often accompanied by myelodysplastic syndrome1. All patients had myeloid lineage-restricted somatic mutations in UBA1 affecting the Met41 residue of the protein and resulting in decreased cellular ubiquitylation activity and hyperinflammation. We here describe the clinical disease course of two VEXAS syndrome patients with somatic UBA1 mutations of which one with a mild phenotype characterized by recurrent rash and symmetric polyarthritis, and another who was initially diagnosed with idiopathic multicentric Castleman disease and developed macrophage activation syndrome as a complication of the VEXAS syndrome. The latter patients was treated with anti-IL6 therapy (siltuximab) leading to a resolution of systemic symptoms and reduction of transfusion requirements.

## Introduction

Recently, a novel disorder named VEXAS (vacuoles, E1 enzyme, X-linked, autoinflammatory, somatic) syndrome was identified in patients with adult-onset inflammatory syndromes, often accompanied by myelodysplastic syndrome (MDS) (1). All patients had myeloid lineage-restricted somatic mutations in UBA1 affecting the Met41 residue of the protein. This promotes the production of an inactive isoform (UBA1c) from a downstream translation site (Met67), resulting in decreased ubiquitylation activity and hyperinflammation (1).

**Figure 1:**
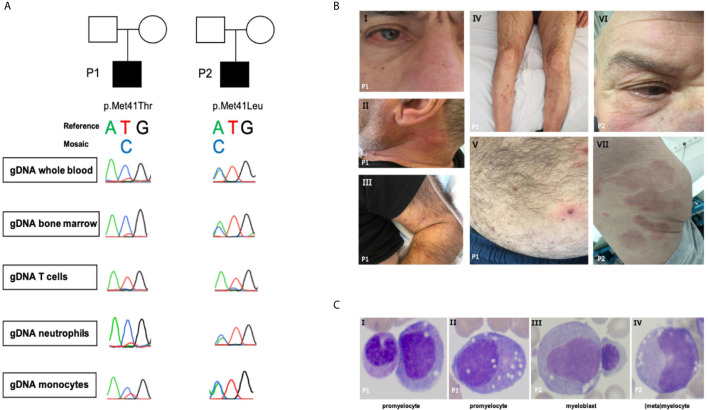
**(A)** Sanger sequence of *UBA1*. **(B.**I) Episcleritis, nose chondritis. **(B.**II) Urticarial lesion of the neck. **(B.**III-V) Nodular lesions on extremities and abdomen: biopsy-confirmed complement-mediated vasculopathy. **(B.**VI) Episcleritis, periorbital erythema **(B**.VII) Urticarial vasculitis of gluteal region. **(C)** Representative aberrant vacuolized bone marrow cells (x40 magnification); left panel also a normal band neutrophil, third panel also a normal erythroblast.

We describe the clinical disease course of two VEXAS syndrome patients (P1 and P2) with two different myeloid lineage-restricted somatic UBA1 mutations (**Figure 1A**). One of these patients was initially diagnosed with idiopathic multicentric Castleman disease (iMCD) and was ultimately treated with siltuximab, a registered IL-6 antagonist.

## Case Description

Patient 1 (**Figure 1A** and **Table 1**) is a 69-year-old male with a medical history of an abdominal hernia for which he had surgery. He presented with 15 kg weight loss over one year, fever up to 38.2° Celsius, night sweats and fatigue since one month, bilateral cervical lymphadenopathy, arthralgia, episcleritis, nose chondritis, thrombophlebitis and nodular lesions (**Figure 1B**.I-V). Laboratory examination showed normocytic anemia (Hb 6.6g/dL, MCV 96fL; reference range 76-96fL), absolute neutrophil count 2.2 x 10^9^/L, thrombocytopenia 46 x 10^9^/L and raised inflammatory markers (CRP 93.1mg/L, normal <5mg/L; ferritin 720µg/L, reference range 30-400µg/L). Cervical lymph node excision biopsy was suggestive of human herpes virus (HHV-)8-negative plasma cell type iMCD and additionally showed hemophagocytosis, while skin nodule biopsy demonstrated subendothelial complement depositions consistent with vasculopathy. Bone marrow examination at diagnosis (**Figure 1C** and **Table 1**) showed trilineage dysplasia with vacuolization and hemophagocytosis (CD68+). Reticulin staining was mildly positive indicating the presence of myelofibrosis (MF grade 1 according to the WHO 2016 classification criteria). Six out of eight hemophagocytic lymphohistiocytosis (HLH)-2004 criteria were fulfilled confirming the diagnosis of HLH. As siltuximab is not reimbursed in Belgium, the patient was treated for iMCD with prednisolone 40 mg and 2 infusions of rituximab (1000 mg) with a time interval of 2 weeks. As corticosteroids could not be tapered and the patient remained transfusion dependent, sirolimus was initiated in second line with good results. However, the patient relapsed quickly after cessation of sirolimus because of toxic concentrations and opportunistic infections. He developed confusion, dyspnea, high grade fever >40°Celsius, complement-mediated vasculopathy with skin ulcerations, anemia (Hb 6.3g/dL, severe thrombocytopenia 3 x 10^9^/L with bleeding complications, and ferritin levels of 3640µg/L (partly due to >28 units red blood cell transfusions). The patient was started on siltuximab (in medical need) and prednisolone 10 mg/day in third line. This resulted in resolution of fever, skin lesions, and normalization of inflammatory markers. From hindsight, serum IL-6 was significantly elevated (102pg/mL, healthy controls <9.5pg/mL, n = 6) prior to siltuximab treatment. Although the patient became less transfusion dependent, his platelets and hemoglobin levels only partially recovered following 2 months of treatment.

**Table 1:** Summary of the demographics, genetic and clinical characteristics, bone marrow examination, and treatment response of the VEXAS patients.

	Patient 1	Patient 2
**Demographic/genetic**		
***Age of onset***	69	75
***UBA1 mutation***	p.Met41Thr	p.Met41Leu
**Clinical spectrum and laboratory findings at presentation**		
***Hematopoietic***	Normocytic anemia	Macrocytic anemia
	Leukopenia	Polyclonal hypergammaglobulinemia
	Lymphopenia	
	Thrombopenia	
***Lymphoproliferation***	Cervical lymphadenopathy	–
	Splenomegaly	
***Constitutional***	Fever	Fever
	Fatigue	Fatigue
	Weight loss	Weight loss
	Night sweats	–
	Raised inflammatory markers	Raised inflammatory markers
	Hyperferritinemia	Hyperferritinemia
***Mucocutaneous***	Panniculitis-like nodular lesions	Urticarial vasculitis
		Livedo reticularis
		Severe skin reaction to anakinra
***Ophthalmologic***	Episcleritis	Episcleritis
***Musculoskeletal***	Arthralgia	Symmetric polyarthritis
	Nose chondritis	Ear chondritis
		Nose chondritis
***Other***	Thrombophlebitis	Deep venous thrombosis
	MAS/HLH	Pulmonary embolism
**Bone marrow examination**	Moderately cellular	Normocellular
	Myeloid vacuolization and dysplasia	Myeloid vacuolization and dysplasia
	Dyserythropoiesis	Dyserythropoiesis
	Micromegakaryocytes	Atypical plasmocytes
	Hemophagocytosis (CD68 +)	Plasmocytosis (6%)
**MDS**		
***Criteria/type***	Not evaluable due to folic acid deficiency	No
***Karyotype***	46, XY[10]	46, XY[10]
***NGS***	DNMT3A (c.1014+1G>T) VAF 31-46%	Negative
**Treatment (response)**		
Corticosteroids*	Prednisone 60mg (+)	Prednisolone 10mg (+)
***Anti-TNF***	–	Infliximab (-)
***Anti-IL-1***	–	Anakinra (-)
***Anti-IL-6***	Siltuximab (+)	–
***Other***	Rituximab (-)	Methotrexate (-)
	Sirolimus (+)	Leflunomide (-)
		Colchicine (-)

^*Minimal dose with clinical response; +, clinical response; -, no clinical response; HLH, hemophagocytic lymphohistiocytosis; MAS, macrophage activation syndrome; MDS,myelodysplastic syndrome; NGS, next generation sequencing.^

Patient 2 (**Figure 1A** and **Table 1**) is a 76-year-old male. His medical history is significant for deep venous thrombosis with pulmonary embolism and type B aortic dissection. He presented with a recurrent rash and a symmetric polyarthritis of both large and small joints (**Figure 1B**.VI–VII). He had no fever and symptoms were rather mild. Laboratory examination showed macrocytic anemia and raised inflammatory markers (CRP 280.9mg/L; ferritin 1161µg/L). Skin biopsy revealed urticarial vasculitis. Bone marrow examination (**Figure 1C** and **Table 1**) showed trilineage dysplasia with myeloid vacuolization and reactive plasmocytosis without evidence of a plasma cell dyscrasia. He was treated with a tapering regimen of corticosteroids, relapsed at a dose of 7.5 mg prednisolone and was subsequently initiated on methotrexate (MTX) 15 mg weekly. Three months later, he developed a bilateral episcleritis for which he received topical indomethacin. Since symptoms were refractory to MTX in combination with prednisolone, anakinra (100 mg daily S.C.) was given for a suspected undifferentiated autoinflammatory syndrome. This resulted in a pronounced, delayed injection site reaction characterized by erythematous infiltrated plaques and subsequent treatment cessation. Considering the inability of tapering the prednisolone below 10 mg daily, anti-TNF-α therapy was initiated. Following the infliximab loading regimen, prednisolone could be reduced to 5 mg daily. Subsequently, the patient developed polyarthritis, a livedoid rash, and polychondritis of the nose and ears, requiring dosage increment to 10 mg prednisolone to remain disease free with subclinical signs of biochemical inflammation.

## Discussion

The two VEXAS patients recently diagnosed within our academic hospital demonstrated a phenotype consistent with the recently described cohort by Beck et al. Both patients had myeloid vacuolization, chondritis and a systemic inflammatory disease state. VEXAS was initially reported to be a highly refractory disease to diverse biologicals (anti-TNF-α, anti-CD20; anti-IL1, anti-IL17 therapy, CTLA4 agonist) only responding to high dose corticosteroids (1). However a recent report on the therapeutic options in VEXAS patients, evaluating the time to next treatment as an objective marker, suggested promising results for the use of JAK inhibitors (ruxolitinib or tofacitinib) in 3 VEXAS patients. One patient under JAK inhibition had markedly regression of cutaneous lesions without increment of corticosteroid dose (2). In neither of the treated patients, an improvement was seen in cytopenia and bone marrow myelodysplastic features. In patient 2, we observed a severe skin reaction to anakinra, which was more pronounced than expected. Severe skin reactions to anakinra were reported by Beck et al. in 62% of patients and may potentially provide a diagnostic clue. Interestingly, patient P1 with relapsing complement-mediated vasculopathy, who responded only to high dose corticosteroid treatment and sirolimus, benefited from siltuximab associated to low dose prednisolone with complete resolution of his inflammatory disease state. After genetic testing revealed VEXAS syndrome, the tentative diagnosis of Castleman disease was reconsidered.

Macrophage activation syndrome (MAS), a form of HLH, is a well-known complication of autoinflammatory disorders (3). MAS in the context of an autoinflammatory condition requires treatment of the underlying disorder, which is challenging in the therapy-refractory VEXAS syndrome (3). The VEXAS syndrome-related inflammatory manifestations in P1 resolved completely following siltuximab treatment. In addition, HLH-criteria were no longer met. This is in contrast with the findings by Beck et al., who reported a lack of response to tocilizumab treatment. Bourbon et al. on the other hand observed some responses to tocilizumab as well as azacytidine, cyclosporine, and JAK inhibitors (2). Siltuximab binds IL-6 itself rather than its receptor as is the case with tocilizumab. Similar unexpected disparities in response were reported previously for infliximab, an anti-TNF-α antibody vs. etanercept, targeting TNF-α as a decoy receptor (4).

In conclusion, the clinical spectrum of somatic UBA1 mutations may vary from a relatively easily low-dose prednisolone-treated VEXAS syndrome to a life-threatening MAS. We extend on the spectrum of cutaneous and hematologic manifestations associated with VEXAS syndrome. Finally, based on our observations, treatment of VEXAS syndrome with siltuximab warrants further evaluation in the absence of evidence-based recommendations. Corroborating the clinical response with additional cytokine measurements before and during treatment could further identify key cytokines and delineate potential targets (1). In line, the pathophysiology of anakinra-induced adverse reactions in VEXAS remains elusive and might benefit from additional cytokine analysis.

## Data Availability Statement

The datasets presented in this study can be found in online repositories. The names of the repository/repositories and accession number(s) can be found in the article/**Supplementary Material**.

## Ethics Statement

The studies involving human participants were reviewed and approved by the ethics committee of University Hospitals Leuven. The patients/participants provided their written informed consent to participate in this study. Written informed consent was obtained from the individual(s), and minor(s)’ legal guardian/next of kin, for the publication of any potentially identifiable images or data included in this article.

## Author Contributions

FS, AlB, SW-A-J, SV, and RS contributed to conception and design of the study. FS and MW performed the experiments for genetic validation and cytokin measurements. SW-A-J and NB performed histopathological evaluation of the bone marrow specimens. AlB, FS, and SW-A-J wrote the first draft of the manuscript. All authors contributed to the article and approved the submitted version.

## Funding

FS (11B5520N) is fellow of the Fonds Wetenschappelijk Onderzoek - Vlaanderen National Fund for Scientific Research (FWO). RS is FWO senior clinical investigator fellows (1805518N, respectively) and received funding from KU Leuven C1 (C12/16/024). SH-B and RS are supported by the VIB Grand Challenge program (Translational science initiative on PID, GC01-C01). DB, SV, and RS are members of the European Reference Network for Rare Immunodeficiency, Autoinflammatory and Autoimmune Diseases (Project ID No 739543).

## Conflict of Interest

The authors declare that the research was conducted in the absence of any commercial or financial relationships that could be construed as a potential conflict of interest.
