# Erratum: Case Report: VEXAS Syndrome: From Mild Symptoms to Life-Threatening Macrophage Activation Syndrome

**DOI:** 10.3389/fimmu.2021.748756

**Published:** 2021-08-10

**Authors:** Frontiers Production Office

**Affiliations:** Frontiers Media SA, Lausanne, Switzerland

**Keywords:** primary immunodeficiencies, autoinflammation, treatment reactions, myelodysplasia, ubiquitination

Due to a production error, an author name was incorrectly written as “Sherida Woei-A-Jin”, which did not match the citation. The correct name is “F J Sherida H Woei-A-Jin”. The publisher apologizes for this mistake.

The original version of this article has been updated.

