# Supplementary material for: Case Report: VEXAS Syndrome: From Mild Symptoms to Life-Threatening Macrophage Activation Syndrome
Source: Front Immunol. 2021 Apr 23;12:678927. doi: 10.3389/fimmu.2021.678927 (PMC8147557; doi:10.3389/fimmu.2021.678927)
Supplement: Supplementary file 1 [file DataSheet_1.docx]

**Methods**

PBMC and granulocyte isolation

Whole blood was diluted 1:1 with RPMI 1640 and layered over lymphocyte separation medium (LSM) (MP Biomedicals, 0850494-CF). Tubes were centrifuged at 400 x g for 25 minutes and PBMCs were harvested and stored in liquid nitrogen until FACS staining. Remaining serum and LSM were discarded, and red blood cells lysed using red blood cell lysis buffer (8.3 g/l NH_4_Cl in 0.01 M Tris–HCl buffer, pH 7.5). The remaining pellet consists of granulocytes which were used for DNA isolation.

FACS

PBMCs were stained in FACS buffer (PBS 1X, 3% FBS) with eBioscience™ Fixable Viability Dye eFluor™ 780 (Invitrogen, 65-0865-14) and fluorochrome-conjugated antibodies direct against human CD3 APC (Biolegend, 300412), CD14 BV510 (Biolegend, 301842) and CD56 FITC (Biolegend, 301842). Sorting was done on a Sony MA900 sorter. Gating strategy is shown in Supplementary Figure 1.

DNA isolation and sequencing

The QIAamp DNA Blood Midi Kit (Qiagen, 51183) was used according to the manufacturers’ instructions. gDNA from the patients was isolated from whole blood, bone marrow aspirate, granulocytes, sorted T cells (CD14^-^CD3^+^CD56^-^) and monocytes (CD14^+^). The amount of gDNA extracted from NK cells was insufficient to perform PCR. The PCR for UBA1 was done using the following primers: forward GCTCCACTCCTGTGTGTCTC and reverse GGGGGTACTCTAGGTCAGGG. Sanger sequencing was done by Eurofins Genomics (Belgium).

Serum IL-6 measurement

Serum IL-6 levels were quantified using the Human IL-6 Quantikine ELISA Kit (R&D systems, D6050) according to the manufacturer’s instructions. The serum IL-6 levels of six healthy controls were below the detection limit (<9.5 pg/ml).

HLH-criteria

HLH-2004 diagnostic criteria in patient 1 were fever (present), splenomegaly (present), bicytopenia (present), hypertriglyceridemia and/or hypofibrinogenemia (present), hemophagocytosis (present), low/absent NK-cell-activity (not determined), hyperferritinemia (present), and high soluble interleukin-2-receptor (not determined), rendering a score of 6/8.
