# Supplementary figures and images for: Case Report: VEXAS Syndrome: From Mild Symptoms to Life-Threatening Macrophage Activation Syndrome
Source: Front Immunol. 2021 Apr 23;12:678927. doi: 10.3389/fimmu.2021.678927 (PMC8147557; doi:10.3389/fimmu.2021.678927)

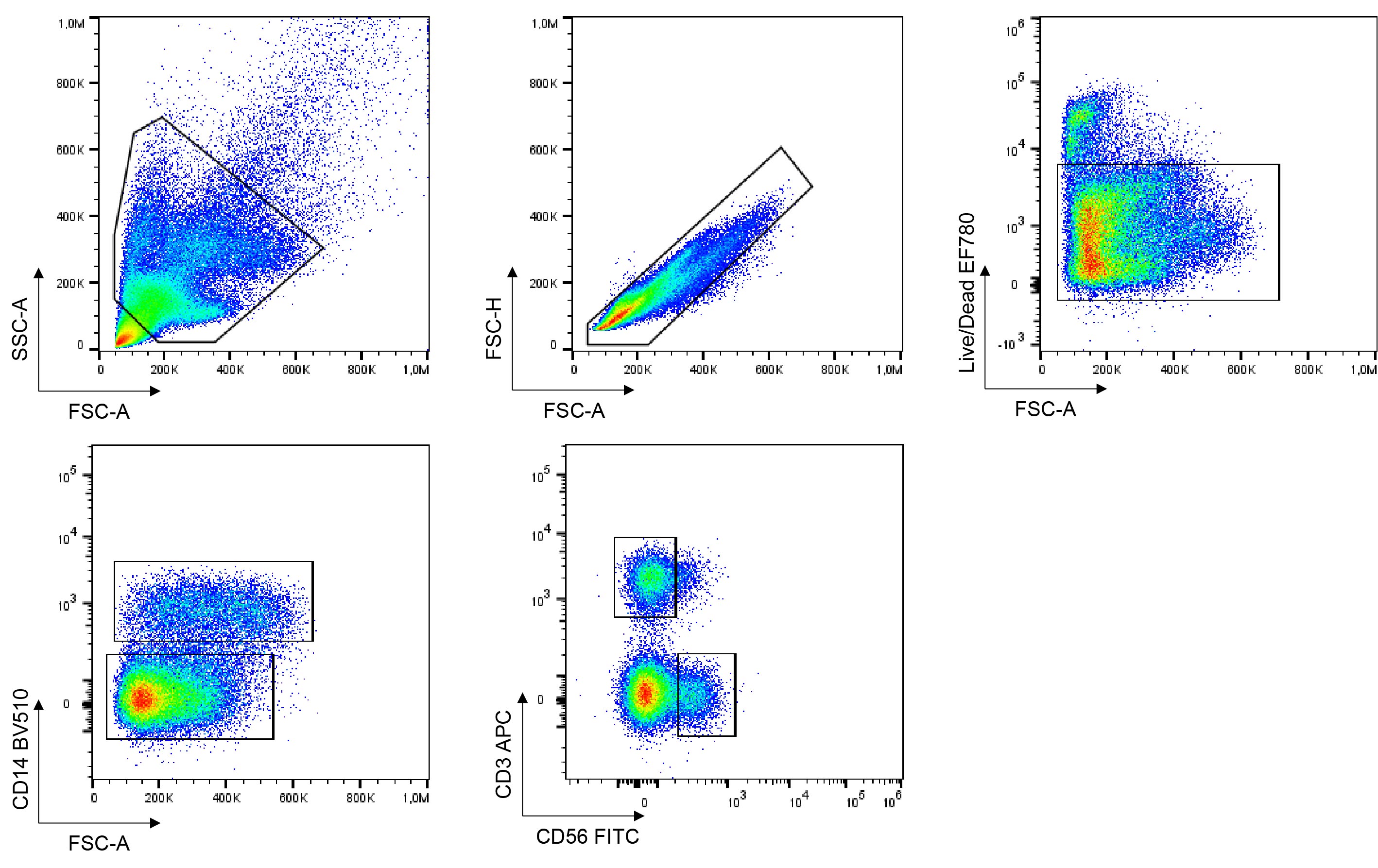

Supplement: Supplementary file 2 [file Image_1.jpeg]
